# S-Adenosyl-L-Methionine Augmentation in Patients with Stage II Treatment-Resistant Major Depressive Disorder: An Open Label, Fixed Dose, Single-Blind Study

**DOI:** 10.1155/2013/204649

**Published:** 2013-05-12

**Authors:** Domenico De Berardis, Stefano Marini, Nicola Serroni, Gabriella Rapini, Felice Iasevoli, Alessandro Valchera, Maria Signorelli, Eugenio Aguglia, Giampaolo Perna, Anatolia Salone, Giuseppe Di Iorio, Giovanni Martinotti, Massimo Di Giannantonio

**Affiliations:** ^1^Chair of Psychiatry, Department of Neurosciences and Imaging, University “G. D'Annunzio”, 66100 Chieti, Italy; ^2^National Health Service (NHS), Department of Mental Health, Psychiatric Service of Diagnosis and Treatment, Hospital “G. Mazzini”, p.zza Italia 1, ASL 4, 64100 Teramo, Italy; ^3^Laboratory of Molecular Psychiatry and Psychopharmacotherapeutics, Section of Psychiatry, Department of Neuroscience, University School of Medicine “Federico II”, 80131 Naples, Italy; ^4^Hermanas Hospitalarias, FoRiPsi, Villa S. Giuseppe Hospital, 63100 Ascoli Piceno, Italy; ^5^Institute of Psychiatry, University of Catania, 95121 Catania, Italy; ^6^Hermanas Hospitalarias, FoRiPsi, Department of Clinical Neurosciences, Villa San Benedetto Menni, Albese con Cassano, Como, Italy; ^7^Department of Psychiatry and Behavioral Sciences, Leonard Miller School of Medicine, University of Miami, Miami, 33124 FL, USA; ^8^Department of Psychiatry and Neuropsychology, University of Maastricht, 6200 MD Maastricht, The Netherlands

## Abstract

We investigated the efficacy of S-Adenosyl-L-Methionine (SAMe) augmentation in patients with treatment-resistant depressive disorder (TRD). Thirty-three outpatients with major depressive episode who failed to respond to at least 8 weeks of treatment with two adequate and stable doses of antidepressants were treated openly with fixed dose of SAMe (800 mg) for 8 weeks, added to existing medication. The primary outcome measure was the change from baseline in total score on Hamilton Rating Scale for Depression (HAM-D). The Clinical Global Impression of Improvement (CGI-I) was rated at the endpoint. Patients with a reduction of 50% or more on HAM-D total score and a CGI-I score of 1 or 2 at endpoint were considered responders; remission was defined as a HAM-D score ≤7. Secondary outcome measures included the Snaith-Hamilton Pleasure Scale (SHAPS) and the Sheehan Disability Scale (SDS). At 8 weeks, a significant decrease in HAM-D score was observed with response achieved by 60% of the patients and remission by 36%. Also a statistically significant reduction in SHAPS and SDS was observed. Our findings indicate that SAMe augmentation may be effective and well tolerated in stage II TRD. However, limitations of the present study must be considered and further placebo-controlled trials are needed.

## 1. Introduction

Major depressive disorder (MD) is a severe, highly prevalent illness that has a substantial impact on public health and human functioning worldwide [1]. Although a large number of effective antidepressant drugs exist, it is well known that a significant proportion of patients with MD fail to achieve response and/or remission with standard antidepressant therapies, even when optimally delivered [2]. Such a condition is called treatment-resistant (or refractory) depression (TRD) and represents a major challenge in everyday “real world” clinical practice [3]. TRD can be classified into different stages based on the degree of resistance. Following the classification of Thase and Rush [4], the stage I treatment-resistant depression is the persistence of significant depressive symptoms, despite at least one adequate trial with one major class of antidepressant, stage II is the stage I resistance plus failure of an adequate trial with an antidepressant in a different class from that used in stage I, whereas stage III is stage II resistance plus failure of an adequate trial with a tricyclic antidepressant.

One of clinical strategies in TRD is to add a second agent with recognized or reputed antidepressant properties [1, 5]. S-Adenosyl-Methionine (commonly known as SAMe) is a naturally occurring molecule distributed in virtually all body tissues and fluids and is involved in many important processes [6]. SAMe plays a role in the immune system, preserves cell membranes, and helps produce and metabolize several brain substances, such as acetylcholine, melatonin, and dopamine. It works with vitamin B12 and vitamin B6 [7]. Being deficient in either vitamin B12 or vitamin B6 may reduce levels of SAMe in the body leading to the development of depressive symptoms [8, 9]. SAMe is necessary for the synthesis and maintenance of a number of neurotransmitters that are involved in the pathophysiology of MD, including serotonin, noradrenaline, and dopamine and this may partially explain its antidepressant properties [10]. Efficacy of SAMe monotherapy in the treatment of MD has been demonstrated in several studies (for an extensive review, see Papakostas et al. [11]). SAMe has been also studies as an adjunctive therapy in patients who failed or were partial responders to selective serotonin reuptake inhibitors (SSRIs) or serotonin norepinephrine reuptake inhibitors (SNRIs) with positive results [12, 13] and has been found to accelerate symptom improvement when given as intramuscular injection in adjunction to imipramine [14]. 

However, according to the classification of Thase and Rush, in the above-mentioned augmentation trials, the majority of the patients belonged to stage I and, to date, efficacy of adjunctive SAMe in stage II TRD has not yet been fully elucidated. Therefore, the aim of this study was to evaluate the use of fixed-dose oral SAMe augmentation in a sample of patients with stage II TRD, assessing efficacy and tolerability of this treatment when added to current antidepressant therapy.

## 2. Methods

Twenty-five adult outpatients (11 males, 14 females) with a DSM-IV diagnosis of MD were recruited from several mental health facilities in Central Italy and referred to our Institute of Psychiatry. All patients gave their written full informed consent to participate in the study prior to enrollment. Diagnoses were made by psychiatrists with at least 5-year clinical experience and supervised by senior psychiatrists (DDB, GM, MDG), following the Structured Clinical Interview for DSM-IV (SCID) [15]. The patients had failed to respond to at least 8 weeks of treatment with two adequate and stable dose of antidepressants (of different classes), as reflected by a baseline Hamilton Depression Rating Scale 21-item version (HAM-D) [16] of 16 or greater and therefore were classified as Stage II treatment-resistance as suggested by Thase and Rush. The concurrent medications at baseline were venlafaxine (*n* = 10, mean dosage = 285.0 mg/d, range 225–375 mg/day); escitalopram 20 mg/day (*n* = 4); duloxetine 120 mg/day (*n* = 3); bupropion 300 mg/day (*n* = 3); agomelatine 50 mg/day (*n* = 3); sertraline 225 mg/day (*n* = 1); mirtazapine 45 mg/day (*n* = 1). At the baseline visit, patients must have been taking a stable dose of an antidepressant for at least 6 weeks.

The exclusion criteria were any other axis I diagnosis (to exclude a diagnosis of bipolar disorder the patients and their first degree relatives were interviewed prior enrollment: a thorough history also was taken to explore bipolar disorder in their other family members and a positive family history was considered as an exclusion criterion), women who were pregnant, nursing, or using inadequate contraception; patients who met DSM-IV criteria for abuse or dependence on any drug including alcohol within 8 months; patients who showed a serious suicide risk during the course of the study; patients with medical contraindications to therapy with SAMe based on medical history and laboratory data; patients with a known allergy or hypersensitivity to SAMe and patients judged by investigators to be unable or unlikely to follow the study protocol. Furthermore, patients were not eligible for the study if they were taking other psychoactive medications or had received electroconvulsive therapy within the 6 months before the initial assessment. Race and gender were not used as a basis for patient selection. 

Concurrent Cognitive-Behavioral (CBT), psychoanalytic, or supportive therapy was not allowed or administered during study period. Patients were forbidden to take any new psychotropic medications during the study. These included benzodiazepines, barbiturates, narcotics, or herbal supplements with presumed psychotropic or analgesic effects. 

Primary outcome measure was the HAM-D total score. The Clinical Global Impression of Improvement (CGI-I) [17] was rated at endpoint. Assessments were carried out at the baseline visit and every week of active treatment until endpoint. Patient raters were blind to the pharmacological treatment of study participants. Secondary outcome measures included the Snaith-Hamilton Pleasure Scale (SHAPS) [18] to assess anhedonia and the Sheehan Disability Scale (SDS) to assess disability: both scales were collected at baseline and endpoint.

SAMe was administered in the following fashion: fixed dose of 800 mg/day in divided doses (morning and afternoon) until study completion (8 weeks). 

Patients with a reduction of 50% or more on the HAM-D total score and a CGI-I score of 1 (very much improved) or 2 (much improved) at endpoint were considered responders to treatment; remission, which represents complete or near complete symptom resolution including resolution of functional impairment, was defined as HAM-D total score of ≤7 [19].

The incidence of spontaneously reported or observed adverse events was reported at every follow-up visit and patients were excluded from the study if side effects were considered as intolerable. At baseline and at the end of the study period, patients underwent blood and urine tests for monitoring of possible changes in vital parameter.

### 2.1. Statistical Analysis

An intent-to-treat analysis was utilized with the last available evaluation carried forward as endpoint (last observation carried forward, LOCF). All observations were collected by patient examiners blind to the pharmacological treatment of study participants. Descriptive statistics (means and standard deviations as appropriate) and percentages were computed for the study sample on demographic variables and all psychometric scales. The data were checked for deviations from the Gaussian distribution using the Kolmogorov-Smirnov test. Analysis of variance for repeated measures (ANOVA) with Bonferroni's post hoc test was used to evaluate HAM-D scores, whereas Student *t* test was used to compare SHAPS and SDS scores between baseline and endpoint. All statistical tests were two-tailed, and statistical significance was declared at the 0.05 *α* level. All results are presented as mean ± standard deviation (SD).

## 3. Results

Twenty-four patients completed the 8 weeks of the study. Patients' data are expressed in Table 1. One patient dropped out after 4 weeks due to obtaining new employment that required relocation and was included in the LOCF analysis. 

**Figure 1:**
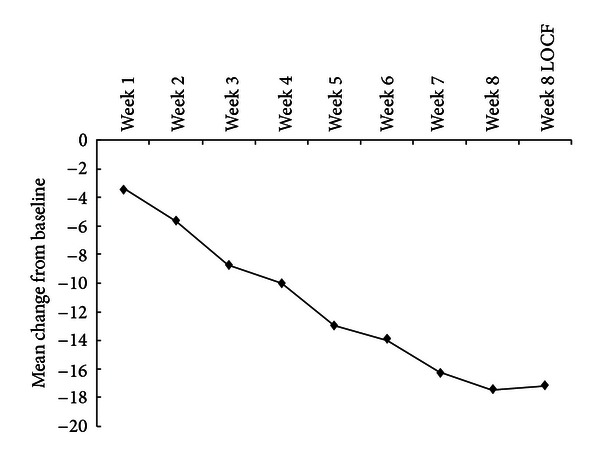
Symptom improvement in patients with TRD on HAM-D.

The decrease in HAM-D scores over time is shown in Figure 1. The mean HAM-D total score at baseline was 28.0 ± 2.9 and reduced to 11.0 ± 5.5 (LOCF) at week 8. A repeated measures ANOVA revealed a significant decrease of scores over time (*F* = 131.3, df = 8, *P* < 0.001, LOCF) with a mean reduction between baseline and endpoint of 17.1 (61.1%). Post hoc test showed that changes from baseline were statistically significant by week 1 (Bonferroni *t* = 4.8, *P* < 0.001). 

Based on a conservative LOCF analysis that included the dropout, at the end of the trial, reduction of more than 50% in HAM-D scores and a score of 1 or 2 on CGI-I was attained in 15 patients (60%; 8 males, 7 females). Ten patients (20.5%; 4 males, 6 females) had a reduction of less than 50% in HAM-D scores and were considered nonresponders. The patient who dropped out was classified as nonresponder. For the LOCF data set, 9 patients (36%; 3 males and 6 females) achieved remission (endpoint score ≤ 7 on HAM-D). The 9 patients who achieved remission from depression came from the group of 15 patients who met criteria of responders.

The effects of SAMe observed on HAM-D were corroborated by the findings for the other efficacy measures. The results are summarized in Table 1 and demonstrate that there were significant changes from baseline for secondary efficacy variables such as SHAPS and SDS (for both scales *P* < 0.001).

In general, side effects were in the mild range and SAMe was well tolerated in the majority of the patients. The most commonly reported adverse events were constipation in six patients (24%) and nausea with decreased appetite in three patients (12%).

## 4. Discussion

To date, the 25 patients included in our study comprise the largest sample of subjects with stage II TRD treated with adjunctive SAMe for an 8-week period. 

Considering that the mean duration of symptoms was 8.0 years, it is apparent that the patients in this study were experiencing long-term illness. In our open trial, results support the notion that adjunctive SAMe may be effective in relieving symptomatology of stage II TRD. A statistically significant clinical improvement was reported within the first week of treatment and continued until the end of the study. Moreover, the majority of responders demonstrated the 50% or more reduction of HAM-D total score within the first 5 weeks of treatment and this means that SAMe augmentation in stage II TRD may be associated with a quite rapid improvement in depressive symptoms. These results were confirmed by significantly improvements seen in all the secondary outcome measures.

The percentage of responders throughout the study was rather high. The clinical significance of the observed change in HAM-D scores was corroborated by relatively high rates of response and remission. Among patients who completed the study, the response rates were as high as 62.5%. Full remission, in which depressed patients are essentially indistinguishable from healthy subjects, was achieved by 37.5% of completers. It has been reported that the remission rates in stage II TRD varied from 10% to 50% with different treatment strategies [3]. However, the STAR*D (the Sequenced Treatment Alternatives to Relieve Depression) trial in patients with stage II TRD showed only a remission rate of 12.3% and 19.8% in patients switched to a third step of treatment option, respectively, mirtazapine or nortriptyline [20]. Recently, a study that evaluated the efficacy and safety of augmenting paroxetine with risperidone, buspirone, valproate, trazodone, or thyroid hormone in stage II TRD patients showed a mean remission rate of 37.3% [21] and these results are in line with those of our study. Moreover, always in terms of response and remission rates, results of our study are similar to what found in patient with stage I TRD augmented with SAMe. In fact, Alpert et al. [12] obtained a response rate of 50% and a remission rate of 43% following augmentation with SAMe in an open trial on 30 patients partial and non-responders to SSRIs or venlafaxine. More recently, Papakostas et al. [13] conducted a randomized, double-blind, placebo-controlled adjunctive trial of SAMe in patients with MD who had failed a prior SSRI trial and found that SAMe was an effective, relatively well-tolerated, and safe adjunctive treatment strategy for SSRIs non-responders patients with MD with response and remission rates higher for patients treated with adjunctive SAMe (36.1% and 25.8%, resp.) than placebo. 

All considered, our results suggest that SAMe augmentation at 800 mg/day may be an option also for the treatment of stage II TRD patients, taking into account also the favourable side-effect profile of such combination. In fact, the side-effect profile of SAMe augmentation was very favourable in our study and quite different from other augmentation strategies [8]. The most frequently observed side effects were constipation and nausea with decreased appetite, both in the mild range. Moreover, the percentage of dropouts was very low for an 8-week study and only one male patient (4%) discontinued the trial after 4 weeks of treatment, not as a consequence of intolerable side effects, but due to obtaining new employment that required relocation. 

There were several limitations to the current study. The four major limitations of our study were the relatively small sample, the open label design, the lack of placebo or active control groups, and the short treatment period (8 weeks). The lack of placebo or active control groups and the open label design may have led to some potential biases such as observer bias, but we tried to reduce eventual halo effects by keeping patient raters blind to the pharmacological treatment of study participants, as made in an our previous study [22]. Moreover, it is not possible to exclude the possibility that the relative high response rate should be partially driven by the placebo response rate, even if stage II TRD is less likely to show a placebo effect [16]. Therefore, further work with SAMe is warranted in placebo-controlled, randomized, double-blind clinical trials on larger samples to assess relative benefits and tolerability. It should also be noted that this was a fixed dose study and no information is available on the optimal dose of SAMe in stage II TRD. Furthermore, long-term studies are required to demonstrate that patients with stage II TRD can benefit from maintenance treatment with such augmentation and to evaluate long-term tolerability.

In conclusion, SAMe augmentation of existing medication may be effective and relatively well tolerated in Stage II TRD patients and future randomized controlled trials are needed to confirm the efficacy and safety of such augmentation strategy.

## Figures and Tables

**Table 1:** Demographic and clinical data of study participants.

Parameter	Baseline	8 weeks (LOCF)	Reduction from baseline (%)	Statistics
Gender				
Male, *n* (%)	11 (43.2%)	—	—	—
Female, *n* (%)	14 (56.8%)	—	—	—
Age, years: mean (SD)	32.0 (5.1)	—	—	—
Age at onset, years: mean (SD)	25.0 (5.1)	—	—	—
Duration of illness, years: mean (SD)	8.0 (2.8)	—	—	—
Experience of hospitalizations: yes, *n* (%)	9 (36%)	—	—	—
History of suicidal attempt: yes, *n* (%)	6 (24%)	—	—	—
HAM-D total score: mean (SD)	28.0 (2.9)	10.9 (5.5)	61.1	*F* = 131.3, df = 8, *P* < 0.001
SHAPS total score: mean (SD)	6.9 (2.2)	3.6 (2.5)	52	*t* = 5.0, df = 48, *P* < 0.001
SDS total score: mean (SD)	16.6 (3.2)	6.7 (5.2)	61.6	*t* = 8.2, df = 48, *P* < 0.001

HAM-D: Hamilton Rating Scale for Depression; SHAPS: Snaith-Hamilton Pleasure Scale; SDS: Sheehan Disability Scale (SDS).
